# Literature review of the portrayal of dentists and teeth in movies

**DOI:** 10.3389/froh.2025.1584382

**Published:** 2025-04-24

**Authors:** Andy Wai Kan Yeung

**Affiliations:** Oral and Maxillofacial Radiology, Applied Oral Sciences and Community Dental Care, Faculty of Dentistry, The University of Hong Kong, Hong Kong, Hong Kong SAR, China

**Keywords:** dentistry, movies, portrayal, content analysis, dental anxiety

## Abstract

**Introduction:**

There are multiple portrayals of dentists and teeth in movies. It is largely unclear if the literature has investigated these portrayals. This literature review aimed to identify and analyze the themes, narratives, and symbolic meanings associated with dental portrayal in movies from the existing literature.

**Methods:**

Online literature databases, Web of Science, Scopus, and PubMed, were searched to identify relevant papers labelled as articles or reviews. Publications were included if they described or investigated the portrayal of dentists or teeth in multiple commercial movies. Publications were excluded if they were irrelevant to this topic or provided commentary on a single movie only. Finally, 7 publications were identified and reviewed. For each publication, its study design, data source, and genres of movies analyzed were recorded. Key findings were reviewed, such as themes or stereotypes identified, positive vs. negative portrayals, and any impact on public perception elaborated.

**Results:**

These papers were published between 2007 and 2024. Five of them provided qualitative content analysis, whereas 2 provided quantitative content analysis. Three publications consulted Internet Movie Database (IMDb) to identify relevant movies. Many of the publications did not explicitly report very detailed inclusion and exclusion criteria for the selection of movies. The portrayal of dentists was covered in 5 publications. The symbolic meaning of teeth was covered in 2 publications. (One publication covered both dentist portrayal and teeth symbolism.) Scenes of oral hygiene practice were investigated in 1 publication.

**Conclusion:**

The portrayal of dentists in movies has often been negative, which may influence public perception and contribute to dental anxiety. Future research should investigate the impact of these portrayals on audience attitudes and behavior, as this review underscores the need for more empirical studies in this area. Meanwhile, readers should notice that one major limitation of this review is the small number of publications included.

## Introduction

The portrayal of professions in media has a profound influence on public perception and societal attitudes (1, 2). One famous theory is the cultivation theory founded by George Gerbner. It posits that television (a common form of media) makes specific and measurable contributions to viewers' conceptions of reality, particularly that increased exposure to television content with recurring messages and images will cultivate shifts in individuals' perceptions (3, 4). Among these professions, dentistry occupies a unique position, often depicted with a mix of fascination and fear. The representation of dentists and dental practices in movies may serve as a powerful medium through which societal attitudes towards oral health and dental professionals are shaped. Despite its potentially significant role in shaping public opinion, the portrayal of dentists and dental practices in movies remains a relatively underexplored area in academic literature.

The depiction of dentists in movies has a long and complex history. From early cinematic portrayals to contemporary representations, dentists have often been depicted in a negative light (5). These portrayals range from the sadistic dentist to the incompetent practitioner. Such negative depictions are not merely artistic choices; they reflect and also spread the long-studied fear and anxiety associated with dental care (6). The early days of media such as television, in particular, were characterized by negative portrayals that contributed to dental anxiety and reinforced stereotypes about dental professionals (7). It might be an issue as dental anxiety is such a popular topic among the public that there are many YouTube videos discussing it (8).

In addition to the portrayal of dentists themselves, scenes involving teeth carry a range of symbolic meaning. Teeth, as potent visual motifs, evoke a range of emotions and associations. In some contexts, teeth symbolize power and strength (9). In other contexts, teeth are associated with intimacy and vulnerability, often depicted in scenes of personal transformation or close interaction (10). Teeth can also evoke horror and disgust, particularly in the horror genre, where dental imagery is used to elicit fear and unease (11). These varied symbolisms underscore the complexity of dental imagery and its potential impact on audiences.

This literature review aimed to provide a comprehensive evaluation of the existing literature on the portrayal of dentists and teeth in movies. By consolidating findings from multiple studies, this review would provide a comprehensive overview of how dentists have been portrayed in movies, highlighting common themes and patterns. Furthermore, by highlighting the methodological gaps and limitations in the existing literature, this review could make recommendations for future research studies that will contribute to a more detailed and evidence-based understanding of this topic. The first objective was to collect and synthesize findings from studies that have examined the portrayal of dentists in movies, providing a comprehensive overview of the current state of research. The second objective was to identify recurring themes, stereotypes, and narratives in the portrayal of dentists. The third objective was to assess the methodologies and theoretical frameworks used in these studies to understand the strengths and limitations of the current research landscape.

## Materials and methods

This literature review adhered to reporting standard of the Preferred Reporting Items for Systematic Reviews and Meta-Analyses (PRISMA) guideline (12) whenever applicable. The protocol for this review was not pre-registered. Ethical approval was not required as it did not involve human or animal subjects.

### Search strategy

On 7 February 2025, online literature databases, Web of Science and Scopus, were queried with the following search string: (dental OR dentist* OR teeth OR tooth) AND (movie* OR “motion picture*” OR cinema*). The term “film” was intentionally omitted in the search string, because a pilot search with this term resulted in many irrelevant research studies on biofilm or radiographic film. The search was limited to the title, abstract, and keyword fields of indexed publications. The search was filtered for articles or reviews only, meaning that other document types such as letters or editorials were excluded. Complementary hand searching of the literature was conducted but identified no additional relevant publications. In response to reviewers' comments, an additional PubMed search was performed on 26 March 2025 with the same search string. Since PubMed does not index keywords and assigns document types in a diverse manner, the PubMed search was limited to the title and abstract fields of indexed publications, without applying any initial filter for document type. Letters or editorials were manually excluded afterwards. The search process yielded 450 unique publications after de-duplication for subsequent screening.

### Inclusion/exclusion criteria

**Figure 1:**
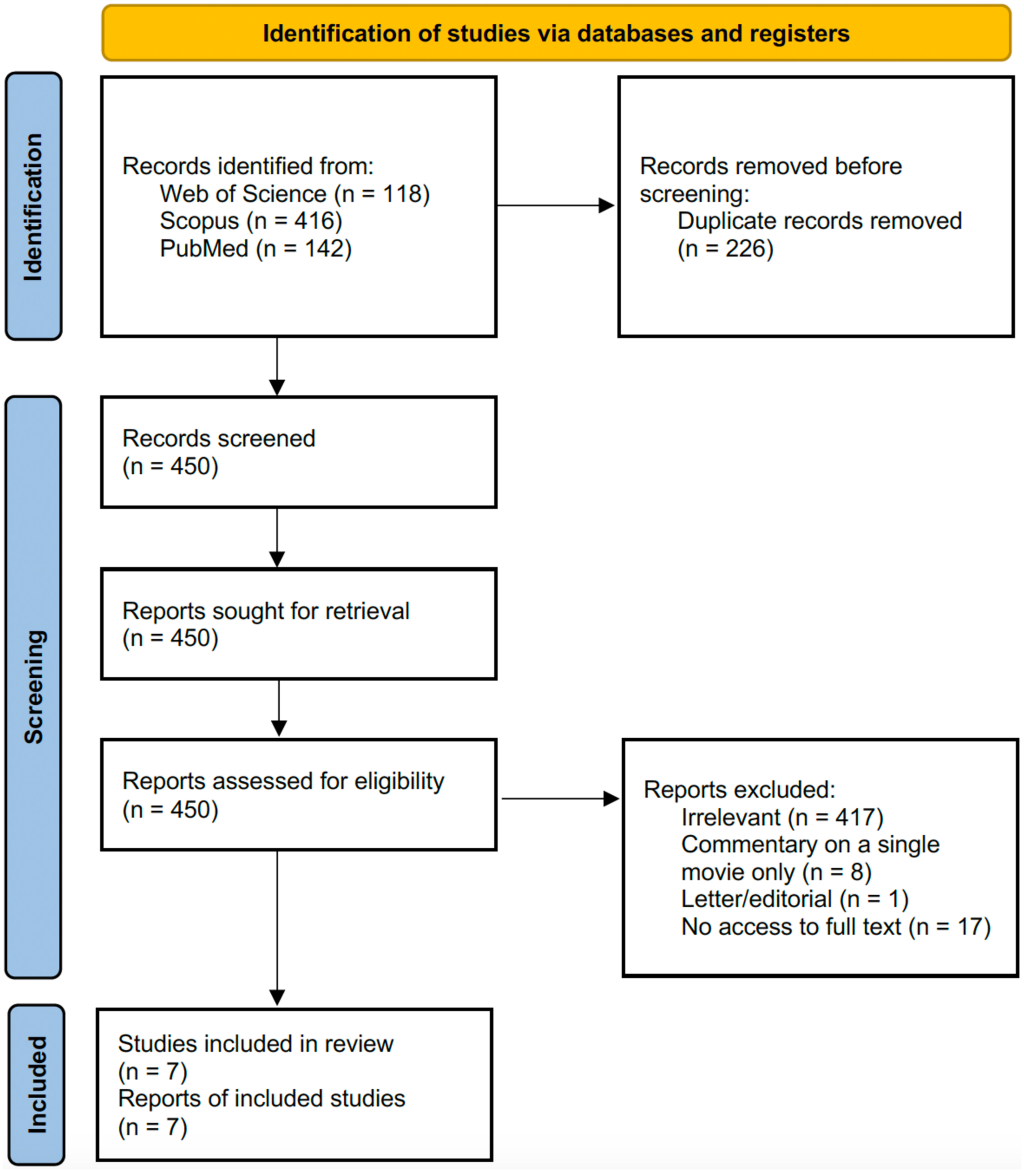
PRISMA flow diagram showing the literature screening process.

The title and abstract of the yielded publications were screened by the author. Publications were included if they described or investigated the portrayal of dentists or teeth in multiple commercial movies. Publications were excluded if they were irrelevant to this topic (e.g., reporting the production of educational videos or short movies for academic purposes, or evaluating “movies” of dynamic medical imaging) or provided commentary on a single movie only. Publications were also excluded if the author had no access to its full text. Finally, 7 publications remained for the review (Figure 1).

### Data extraction

For each included publication, basic bibliographic characteristics were recorded, such as its study design, data source, and genres of movies analyzed. Key findings were noted, such as themes or stereotypes identified, positive vs. negative portrayals, and any impact on public perception elaborated. Since the reviewed publications were content analysis on movies instead of ordinary narrative or systematic reviews on journal articles, there seemed to be no established tool for a quality assessment or risk of bias assessment so that such assessment was not performed. The literature screening and data extraction were performed by a single author, without the use of automation tools.

## Results

The basic bibliographic characteristics of the 7 included publications are listed in Table 1. These papers were published between 2007 and 2024. Five of them provided qualitative content analysis, whereas 2 provided quantitative content analysis. Three publications consulted Internet Movie Database (IMDb) to identify relevant movies, whereas 2 publications consulted multiple databases. Another 2 publications did not specify how they identified the relevant movies. With regard to the genres of movies covered, 5 publications included all genres, one publication focused on tap dancing, and the remaining one publication focused on animated movies. Many of the publications did not explicitly report very detailed inclusion and exclusion criteria for the selection of movies. Each of the 7 publications reviewed 2–137 movies, with a median of 33. The portrayal of dentists was covered in 5 publications. The symbolic meaning of teeth was covered in 2 publications. (One publication covered both dentist portrayal and teeth symbolism.) Scenes of oral hygiene practice were investigated in 1 publication. The main take-home messages from these publications, though not entirely unanimous, are that dentists are often portrayed negatively in movies, whereas teeth are depicted with various symbolic implications.

**Table 1:** Summary of identified studies.

Publication	Study design	Data source	Genres of movies analyzed	Inclusion/exclusion criteria	Number of movies with dentists or teeth	Major take-home message
Thibodeau et al. (13)	Qualitative content analysis	IMDb	All	Unclear	137	Dentists are often portrayed in a comedic role or as incompetent, sadistic, immoral, disturbed or corrupt.
						A notable trend is the inclusion of historically underrepresented groups.
Morrison (17)	Qualitative content analysis	Unclear	Tap dancing	Unclear	2	Scenes showing virtuosic footwork were intercut with close-ups of toothy grins that suggested power, intimacy, and submission.
Gierok et al. (5)	Qualitative content analysis	Google, IMDb, Moviepilot.de, website of Dr Felix Schiminke dental practice^a^	All	Inclusion: Depicting a dental character, a patient and a dental examination and/or treatment; released during 1913–2013; produced in the United States	55	The portrayal of dentists has changed from the figure of “Dr Awkward” in the silent film era, “Dr Prosperous” in the 1960s/1970s and “Dr Evil” in the 1980s/1990s.
				Exclusion: Documentaries, children's movies, TV movies		
Kitsaras et al. (20)	Quantitative content analysis	IMDb	Animated movies	Inclusion: movies of at least 40 min; top 30 highest grossing of all time	30	Toothbrushing and flossing were shown in 5 and 1 scene only, respectively. In contrast, sugar intake was shown 74 times.
Simpson et al. (22)	Quantitative content analysis	IMDb, Wikipedia, previously published papers (5, 13, 28)	All	Inclusion: Top 100 grossing during their year of release; released during 1980–2019; contained a character who is a dental professional or impersonates a dentist, depicted dental treatment or procedures, or contained a scene of tooth extraction or oral mutilation even if not performed by a dentist	60	Many portrayals of dentists were negative (50%) instead of positive (5%). Half of the movies contained scenes of clinical dentistry, which were often medically inaccurate (30% of 30 movies). 57% of patients in clinical scenes were distressed or physically harmed.
				Exclusion: Released via home videos or streaming services; only had characters wearing orthodontic appliances or scenes of untreated dental trauma		
Vidal (23)	Qualitative content analysis	IMDb	All	Inclusion: Representative examples from 384 movies identified from the search as determined by Vidal	33	The image of dentists has gradually evolved from being caricatures to competent, trusted health professionals.
Marini (11)	Qualitative content analysis	Unclear	All	Inclusion: Movies produced in the United States	27	There are 3 recurring dental horror themes in movies: dental violence (including dentistry and dental torture), dental disease/caries, and the legacy of the tooth fairy folk tale.

IMDb, internet movie database.

^a^ https://www.drschminke.de/dentalfilme.html.

## Discussion

This literature review has identified 7 publications that provided a review or evaluation of movies with scenes of dentistry or teeth. Through the variety of genre and time of release, there was an overall negative perception towards dentistry on the screen. Detailed commentaries on these 7 publications are provided below.

### Critique on Thibodeau and Mentasti (2007)

Thibodeau and Mentasti (13) caught the attention from the audience by stating that it was a dentist who kidnapped the main character, a fish called Nemo, in the movie Finding Nemo (2003). They have identified 137 movies with portrayal of dentists, released from 1911–2007. It was found that >60% of these movies were comedies. However, many of them may build up the comedic tone by sacrificing or distorting the image of dentistry. For instance, in Charlie and the Chocolate Factory (2005), the main character, Willy Wonka, blames his strange behavior on his childhood, during which he was subjected to orthodontic treatment and deprived of candies by his dentist father. Thibodeau and Mentasti has found that since the 2000s more non-white, non-male dentists have been shown in movies to show an improved diversity.

The authors have argued that it is important to counter the negative portrayal of dentistry in the movies with millions of Americans avoiding dental care due to dental anxiety. They cited that a past study (14) to highlight that negative expectation arising from information obtained from friends, relatives and mass media sources including movies and television is one of the most common explanations for dental anxiety. Upon closer examination, the survey conducted by Bernstein et al. in 1979 was actually a secondary source. It only mentioned that “vicarious negative experiences portrayed by family, friends, and the mass media” was one of the 4 main categories focused by prior studies on the origin of dental anxiety, and cited two studies for this particular category of origin (7, 15). Tracing to these primary sources, Kleinknecht et al. (7) reported that “about 17% of the participants noted that stories told by friends and relatives and on television and in cartoons had given them an expectation of trauma from dentists”, whereas Shoben Jr and Borland (15) did not deal with mass media. In other words, the effect of negative portrayal of dentists from televisions and cartoons on public perception towards dentistry was extrapolated to movies by Thibodeau and Mentasti. A similarly untested claim can be found for the declining popularity of mahjong parlors in Hong Kong, which were used to be very popular recreational places for Hong Kong people during leisure time. One suggested reason for the decline, though untested, is the perceived association of mahjong parlors with gangsters, a perception created by frequent cinematic scenes depicting gangsters gathering or fighting at mahjong parlors, bars, and nightclubs in the old neighborhoods of Hong Kong (16).

### Critique on Morrison (2014)

Morrison (17) examined the close-up shots of smiles and teeth in tap dancing movies featuring Bill Robinson and Eleanor Powell. She argued that these scenes, intercut with full-body shots of tap dancing, represented complex narratives of power, intimacy, and submission. To begin with, the close-up shots of the dancers' faces established their star power and importance as performers. For example, both Bill Robinson and Eleanor Powell were shown confidently commanding their performance spaces, focusing the attention of their staged audience, and by extension, the live audience. Then, the close-ups created a sense of intimacy between the dancer and the audience. By looking directly into the camera and smiling, the dancers appeared to gaze into the viewers' eyes, making the performance seem personal and directed at each individual viewer. Finally, the smiling close-ups also evoked the sense of submission. For Robinson, these shots could be perceived as a depiction of racial subservience, perpetuating the legacy of minstrelsy in Hollywood by presenting the “smiling minstrel mask” associated with racial stereotypes from the early days. For Powell, the close-ups emphasized her feminine sexual availability, aligning with the voyeuristic tradition of depicting beaming, passive chorus girls in 1930s movies. In short, teeth showing was deemed to be attractive to the audience from several perspectives.

This publication focused on two movies only, The Littlest Rebel (1935) and Broadway Melody of 1936 (1935), so the conclusions made by Morrison might not be readily extrapolated to other Hollywood tap dancing movies at that time. Moreover, Morrison cited multiple books and book chapters to substantiate her claims, but it was highly likely that the references similarly provided a subjective narrative on the topics without conducting scientific studies with hypothesis testing. A Japanese study found that male faces gave a strong impression of sociability and activity when smiling with the teeth exposed, whereas females gave a strong impression of friendliness and elegance with the same grin (18). It partially echoed with Morrison's perspective that a smiling face from a male and a female might create different impressions by the audience, though other claims such as racial stereotype and sense of submission were untested.

### Critique on Gierok et al. (2022)

Gierok et al. (5) examines the portrayal of dentists in American feature films over a century. Interestingly, one of the databases that the authors searched was a website from a private dental practice with a page dedicated to movies and TV dramas with dental components. The study identified 55 movies produced between 1913 and 2013 that featured dental treatment, revealing that dentists appeared mainly in comedies and typically as supporting characters. The study arbitrarily divided the 100-year time span into five 20-year time spans. The portrayal of dental procedures evolved from surgical extractions in early films to more tooth-preserving therapies in later years. During the 1st time span (1913–1932), tooth extraction with ineffective or no anaesthesia was a common plot to produce a crude situation comedy. In the 2nd time span (1933–1956), not only tooth extraction but also conservative dentistry was shown, such as the insertion of dental prosthesis. During this period, there was even a biographical movie called The Great Moment (1944), which told the story of Dr William T. G. Morton, a famous American dentist who discovered the use of ether for general anesthesia. During the 3rd time span (1957–1979), dentists were usually given a minor supporting role. One notable exception was the Marathon Man (1976), in which a dentist-cum-war criminal tortured a man by drilling into his teeth without anaesthesia to extort the information about the location of hidden diamonds. During the 4th time span (1980–1999), dentists shown on screen were gradually wearing personal protection equipment such as gloves and masks. Moreover, inspired by Marathon Man, the dental horror genre was further developed with The Dentist (1996) and The Dentist 2 (1998), in which the main character was a psychotic dentist that conducted violent acts against his patients, effectively transforming the dentist into a source of horror. Gierok et al. acknowledged these two movies as they incorporated accurate and realistic elements of dental practice, such as prophylaxis, radiation protection, dental implants, and aesthetic dentistry. Though Gierok et al. claimed that the first female dentist shown in the movies was in Burglar (1987), it was refuted by Brand and Voorbraak (19) that it should be The Fair Dentist (1911). Finally, during the 5th time span (2000–2013), there were at least 17 dentists in the movies, with the very first African American dentist and a dentist couple according to the authors.

The primary issue with the study by Gierok et al. lies in the casual approach to content analysis, as well as the seemingly arbitrary division of the five time spans into 20-year intervals, which collectively cover 100 years. The changes in the portrayal of dentists in movies were based on the authors' expert opinion, supported by the content of a few representative movies. However, there was no systematic content coding or data extraction evident in their methodology. For instance, the study mentioned that during the fourth time span (1980–1999), dentists on screen were gradually shown wearing personal protection equipment such as gloves and masks. This raises several questions: Does this imply that all dentists in movies from this period were depicted wearing gloves and masks? Or does it indicate that the authors observed the first instance of a dentist wearing such equipment during this time? Without systematic data collection and analysis, it is challenging to draw definitive conclusions from these observations.

A more rigorous approach would involve a detailed content analysis with systematic coding to quantify the presence of specific traits and behaviors of dentists in movies across different time periods. This would provide a more objective and reliable basis for understanding how the portrayal of dentists has evolved over time.

### Critique on Kitsaras et al. (2023)

Kitsaras and Goodwin (20) examined how oral health behaviors were depicted in the top 30 highest grossing animated movies of all time. They aimed to understand if these movies included oral hygiene practices or risk-related behaviors that could influence children's habits and attitudes towards oral health. They identified 100 instances of dental-related behaviors. The majority (93%) of them being risk-related behaviors, mostly involving sugar consumption (74%) followed by drinking alcohol (16%). Oral hygiene practices constituted the minority (7%): 5 instances of toothbrushing, one instance of flossing and one instance of using sippy cup with water. Kitsaras and Goodwin found that both good and evil characters engaged in risk behaviors, with no significant association between the nature of the character and the type of behavior. Meanwhile, human-like characters were more likely to display risk behaviors. Most behaviors were visually depicted (53%), with a smaller proportion being verbal (10%) or a combination of both (37%). The authors concluded that, given the high incidence of risk behaviors portrayed, there was a need for better representation of positive oral hygiene practices in children's media to promote better oral health outcomes.

The point of using sippy cup with water is interesting. It was classified by Kitsaras and Goodwin as an good oral hygiene practice. Perhaps they focused on having water instead of juice or sugary drinks, rendering the behavior healthy to the teeth. On the other hand, the prolonged use of sippy cup was discouraged by some pediatric dentists as they believed that it might promote caries if it contained sugary drinks (21). A recent survey also reported that 40.3% of children used a bottle or sippy cup that contained nonwater beverages, implying that sippy cups could be frequently filled by sugary drinks.

Another point is the list of pre-defined behaviors to be recorded. From their supplementary file, oral hygiene practices contained 4 items, brushing, flossing, using mouthwash, and rinsing. The use of sippy cup with water might be roughly classified as rinsing, but this is only a speculation as the authors did not explain on this. On other other hand, only sugary consumption was present in the list, but not acidic food, including sports drinks, which can also be harmful to the teeth by means of erosion. The main critique here is that eating scenes must be more frequent than scenes showing characters performing oral hygiene practices. To enable a fairer comparison, good vs. bad practices could be made, both of which should contain contextually similar behaviors. For example, for toothbrushing scenes, those showing modified Bass technique should be good practice, whereas those showing mere to-and-fro actions should be bad practice. Considering eating scenes, if sugary consumption is perceived as bad practice, perhaps eating vegetables or non-sugary food can be perceived as good practice.

### Critique on Simpson and Smillie (2023)

Simpson and Smillie (22) analyzed the portrayal of dentistry in movies, classified the scenes according to their potential impact on public perception of dentistry as positive, neutral, or negative, and assessed the accuracy of the clinical scenes depicted. They examined the top 100 grossing movies per year at the US box office from 1980–2019, eventually identifying 60 movies that featured dental professionals, dental treatment, or dental-themed scenes. One of their key findings was that 50% of the movies depicted dentistry negatively, often showing dentists as sadistic, unethical, or involved in crime. Out of 30 movies depicting clinical dentistry, only 23% were accurate, while 30% were exaggerated and 30% were inaccurate. Patients were often shown as distressed or physically harmed in exaggerated clinical scenes, with 8 movies showing dental patients moaning, crying, or grimacing. Recurring themes included crime, sexual misconduct, and an unfavorable comparison with medicine. Sedation was shown or discussed in 11 movies, only 3 of which demonstrated responsible use of it whereas another 6 showed deliberate abuse.

This study was the only one among the 7 publications included in the current review that provided a coded data sheet. The data sheet, provided as a supplementary material by Simpson and Smillie (22), listed the data for each of the 60 analyzed movies. Hence, data transparency is a strength of this study. On the other hand, the analysis would be more comprehensive if more movies, not limited to top 100 grossing movies at the US box office, could be analyzed. Another aspect that could be potentially analyzed is the duration of the dental scenes. A scene that lasts a few seconds with no dialogue may have a much less impact on the audience than a scene that lasts a few minutes with main characters talking or shouting.

### Critique on Vidal (2023)

Vidal (23) identified 384 movies by searching the word dentist from the IMDb database. Vidal found that dentists were depicted as secondary, comical characters in silent films released in early days, often in exaggerated and unflattering ways. Later, dentists began to be portrayed as more complex and central characters. During the 1970s, movies like Marathon Man (1976) depicted dentists as evil characters, contributing to a pure negative image. After that, there was a shift away from this negative portrayal to contribute to a more sophisticated and humanized image of dentists, such as dentists who experienced midlife crisis. Vidal also affirmed the importance of realistic depictions of dental offices and procedures in modern movies, enhancing credibility and reducing dental anxiety among viewers. For instance, instead of repeating the cliché pliers joke (i.e., using outdated or exaggerated pliers to remove tooth), some modern movies would recruit dentists as consultants to ensure a realistic depiction of the dental office, scenes of painless anesthesia, scaling, setting up rubber dam, restoring teeth with composite, and so on.

Similar to prior studies that focused on qualitative content analysis, this study also exhibited subjectivity. As Vidal noted, the study “focus[ed] our attention on some of them, chronologically […] without aiming at exhaustiveness and abandoning de facto objectivity”. A total of thirty-three movies, out of 384, were mentioned and elaborated upon. The remaining majority was omitted. Hence, the narrative might not reflect the temporal change of the movie landscape precisely. Since the identification of the 384 movies was based on a keyword “dentist” without considering box office performance, it is unclear about the audience reach, and hence the potential impact on the public perception, of these movies. It would be more objective if Vidal could elaborate on how the representative examples were chosen from the identified list.

### Critique on Marini (2024)

Marini (11) focused on dental horror movies. She argued that while dental horror is rarely a central theme in movies, it is pervasive and connected to deeper meanings of power, aesthetics, and human integrity. Marini identified three main themes in dental horror: dental violence (including dentistry and dental torture), dental disease and decay, and the legacy of the Tooth Fairy folk tale. For the dental violence theme, it involves scenes of dental torture and violent dental acts showing dentist chairs and dental tools, causing a sense of powerlessness and fear of pain in the victim. Movies such as The Dentist (1996) and its sequel explored the sadistic nature of dental professionals. Non-professional settings also depicted dental torture, as seen in Marathon Man (1976) and Saw 3D (2010). For the theme on dental disease and decay, Marini elaborated on how the loss of teeth signified a character's transformation or decay, both corporeal and psychological. This is often depicted through self-extraction of teeth, marking a pivotal change in the character, such as in Stir of Echoes (1999), A Cure for Wellness (2016), and The Fly (1986), in which the involved character immediately thereafter developed supernatural sense, suspected the toxicity of a cure, and transformed into a monstruous insectoid creature, respectively. Finally, for the theme on the Tooth Fairy legacy, the horror rooted in the folklore of the Tooth Fairy, focusing on the trauma of losing baby teeth and the transition from childhood to adulthood, was exploited. Movies such as Darkness Falls (2003), The Tooth Fairy (2006), and Don't Be Afraid of the Dark (2010) depicted monstrous entities that collected children's teeth, emphasizing a light vs. dark dichotomy.

This study has highlighted an interesting and recurring theme in movies, dental horror. However, it did not refer to psychological theories or research that explain why dental horror was particularly effective or disturbing for viewers, or why viewers enjoyed watching it. In fact, a recent review paper has elaborated on several psychological theories on why people enjoy watching horror movies, namely excitation transfer theory, for uses and gratification, arousal, social comparison, dispositional alignment or empathy, and sensation seeking (24). Readers may refer to (24) for a comprehensive discussion on these topics.

### Portrayal of other professions in movies and other media

A recent study used computational text analysis to examine the portrayal of over 4,000 professions in subtitles from 136,000 movies and TV shows spanning 1950–2017. It was recognized that the sentiment expressed toward lawyers, police, and doctors showed increasing negative trends over time, but the mentions about astronauts, musicians, singers, and engineers are more positive (25). Another study of the portrayal of accountants in 121 movies distributed in North America until year 2000 has found a total of 168 accountant characters who could be classified into 5 stereotypes: dreamer, plodder, eccentric, hero and villain (26). There was an increasing trend in the representation of females, ethnic minorities, certified public accountants and chartered accountants. Meanwhile, a comparative analysis of 670 nurse and 466 physician characters in novels, movies, and television series during 1920–1980 has found that nurses were consistently depicted as less intelligent, less rational, and less central to plots compared to physicians, reinforcing gender and professional hierarchies (27). These examples suggested that the portrayal of dentists in movies can similarly be analyzed in a much more detailed way by future quantitative content analyses, in the style of (22), instead of selecting representative examples for elaborations.

### Limitations of the current review

Readers should notice that one major limitation of this review is the small number of publications included. Also, the review was conducted by a single author. Most of the 17 publications excluded due to no access to full-text were published before 2000. The author was unable to access them through online resources or from the university library.

## Conclusion

In summary, this review has identified and evaluated 7 publications on the portrayal of dentists and teeth in movies. Most of these publications provided a qualitative content analysis in a non-systematic manner. Only two publications offered a quantitative content analysis, with one supplying their coded data for data transparency. The portrayal of dentists in movies was often negative, especially in the early days. Meanwhile, scenes of teeth might carry numerous symbolisms, ranging from power, intimacy, to horror. The dental profession should be consulted to ensure that dentistry is accurately depicted without exaggeration to avoid unnecessary negative publicity. Future studies on the portrayal of dentists in movies should be analyzed in a much more detailed way such as in the style of (22), instead of handpicking representative examples for elaborations. There should also be experiments or surveys to investigate whether these portrayals have any actual impact, positive or negative, on the audience's perception of dentistry.

## Data Availability

The original contributions presented in the study are included in the article/Supplementary Material, further inquiries can be directed to the corresponding author.

## References

[1] Pfau M Mullen LJ Deidrich T Garrow K. Television viewing and public perceptions of attorneys. Hum Commun Res. (1995) 21:307–30. 10.1111/j.1468-2958.1995.tb00349.x

[2] Kagan M Lissitsa S. Generations X, Y, Z: attitudes toward social workers in the age of media technologies. Technol Soc. (2023) 75:102353. 10.1016/j.techsoc.2023.102353

[3] Gerbner G Gross L. Living with television: the violence profile. J Commun. (1976) 26:172–99. 10.1111/j.1460-2466.1976.tb01397.x 932235

[4] Gerbner G Gross L Morgan M Signorielli N. The “mainstreaming” of America: violence profile number 11. J Commun. (1980) 30:10–29. 10.1111/j.1460-2466.1980.tb01987.x

[5] Gierok S Mirza SA Karenberg A. Dentists in action: a profession on-screen (1913–2013). Br Dent J. (2022) 232:737–41. 10.1038/s41415-022-4145-6 35624265 PMC9142361

[6] Yeung AWK. A bibliometric analysis on the early works of dental anxiety. Dent J. (2023) 11:36. 10.3390/dj11020036 PMC9955892 36826181

[7] Kleinknecht RA Klepac RK Alexander LD. Origins and characteristics of fear of dentistry. J Am Dent Assoc. (1973) 86:842–8. 10.14219/jada.archive.1973.0165 4511174

[8] Wong NSM Yeung AWK Mcgrath CP Leung YY. Qualitative evaluation of YouTube videos on dental fear, anxiety and phobia. Int J Environ Res Public Health. (2022) 20:750. 10.3390/ijerph20010750 36613071 PMC9819845

[9] Capps D Carlin N. Sublimation and symbolization: the case of dental anxiety and the symbolic meaning of teeth. Pastoral Psychol. (2011) 60:773–89. 10.1007/s11089-011-0368-1

[10] Kelly CR. Camp horror and the gendered politics of screen violence: subverting the monstrous-feminine in teeth (2007). Women’s Stud Commun. (2016) 39:86–106. 10.1080/07491409.2015.1126776

[11] Marini AM. Tracing dental horror in contemporary American cinema. Horror Stud. (2024) 15:9–24. 10.1386/host_00077_1

[12] Page MJ Mckenzie JE Bossuyt PM Boutron I Hoffmann TC Mulrow CD The PRISMA 2020 statement: an updated guideline for reporting systematic reviews. Br Med J. (2021) 372(71):n71. 10.1136/bmj.n71 33782057 PMC8005924

[13] Thibodeau E Mentasti L. Who stole nemo? J Am Dent Assoc. (2007) 138:656–60. 10.14219/jada.archive.2007.0238 17473045

[14] Bernstein DA Kleinknecht RA Alexander LD. Antecedents of dental fear. J Public Health Dent. (1979) 39:113–24. 10.1111/j.1752-7325.1979.tb02932.x 287803

[15] Shoben EJ Jr. Borland L. An empirical study of the etiology of dental fears. J Clin Psychol. (1954) 10:171–4. 10.1002/1097-4679(195404)10:2<171::AID-JCLP2270100214>3.0.CO;2-O 13143127

[16] Desser D. Triads and changing times: the national allegory of Hong Kong cinema, 1996–2000. Q Rev Film Video. (2009) 26:179–93. 10.1080/10509200902841627

[17] Morrison M. Tap and teeth: virtuosity and the smile in the films of bill Robinson and Eleanor Powell. Dance Res J. (2014) 46:21–37. 10.1017/S0149767714000266

[18] Miyazono M Torii K Yamamoto M Tanaka J Tanaka M. Evaluation of the impression imparted on others by a smile that shows the teeth, using the semantic differential method. J Osaka Dent Univ. (2019) 53:179–86.

[19] Brand H Voorbraak P. Women dentists in films. Br Dent J. (2022) 233:3. 10.1038/s41415-022-4465-6 35804101

[20] Kitsaras G Goodwin M. Portrayal of oral hygiene and risk behaviours in animated movies. Front Oral Health. (2023) 4:1116717. 10.3389/froh.2023.1116717 37475981 PMC10354335

[21] Tinanoff N Palmer CA. Dietary determinants of dental caries and dietary recommendations for preschool children. J Public Health Dent. (2000) 60:197–206. 10.1111/j.1752-7325.2000.tb03328.x 11109219

[22] Simpson CD Smillie SM. An analysis of the portrayal of dentistry in modern popular film from a dentist’s perspective. Br Dent J. (2023) 235:421–5. 10.1038/s41415-023-6268-9 37737414

[23] Vidal C. Evolution of the representation of the dentist in cinema. J Med Movies. (2023) 19:53–60.

[24] Martin GN. (Why) do you like scary movies? A review of the empirical research on psychological responses to horror films. Front Psychol. (2019) 10:2298. 10.3389/fpsyg.2019.02298 31681095 PMC6813198

[25] Baruah S Somandepalli K Narayanan S. Representation of professions in entertainment media: insights into frequency and sentiment trends through computational text analysis. PLoS One. (2022) 17:e0267812. 10.1371/journal.pone.0267812 35584111 PMC9116627

[26] Dimnik T Felton S. Accountant stereotypes in movies distributed in North America in the twentieth century. Account Organ Soc. (2006) 31:129–55. 10.1016/j.aos.2004.10.001

[27] Kalisch PA Kalisch BJ. A comparative analysis of nurse and physician characters in the entertainment media. J Adv Nurs. (1986) 11:179–95. 10.1111/j.1365-2648.1986.tb01236.x 3635547

[28] Brand H. Dentists on film: the monuments men. Br Dent J. (2018) 224:670. 10.1038/sj.bdj.2018.372 29747187

